# How water availability influences morphological and biomechanical properties in the one-leaf plant *Monophyllaea horsfieldii*

**DOI:** 10.1098/rsos.171076

**Published:** 2018-01-03

**Authors:** Tim Kampowski, Max David Mylo, Simon Poppinga, Thomas Speck

**Affiliations:** 1 Plant Biomechanics Group Freiburg, Botanic Garden, University of Freiburg, Freiburg im Breisgau, Germany; 2 Freiburg Materials Research Center, University of Freiburg, Freiburg im Breisgau, Germany

**Keywords:** biomechanics, functional morphology, *Monophyllaea*, *Ramonda*, turgor pressure, drought tolerance

## Abstract

In its natural habitat, the one-leaf plant *Monophyllaea horsfieldii* (Gesneriaceae) shows striking postural changes and dramatic loss of stability in response to intermittently occurring droughts. As the morphological, anatomical and biomechanical bases of these alterations are as yet unclear, we examined the influence of varying water contents on *M. horsfieldii* by conducting dehydration–rehydration experiments together with various imaging techniques as well as quantitative bending and turgor pressure measurements. As long as only moderate water stress was applied, gradual reductions in hypocotyl diameters and structural bending moduli during dehydration were almost always rapidly recovered in acropetal direction upon rehydration. On an anatomical scale, *M. horsfieldii* hypocotyls revealed substantial water stress-induced alterations in parenchymatous tissues, whereas the cell form and structure of epidermal and vascular tissues hardly changed. In summary, the functional morphology and biomechanics of *M. horsfieldii* hypocotyls directly correlated with water status alterations and associated physiological parameters (i.e. turgor pressure). Moreover, *M. horsfieldii* showed only little passive structural–functional adaptations to dehydration in comparison with poikilohydrous *Ramonda myconi*.

## 1. Background

In contrast to the neighbouring rainforest ground flora, Malaysian limestone outcrops are characterized by higher wind, rain and light exposures and by periodic low water availabilities due to thin, rapidly draining soil layers [1,2]. Oftentimes, these habitats are inhabited by members of the Gesneriaceae family (gesneriads), such as *Monophyllaea horsfieldii* R.Br. [1,3]. This species is predominantly known for its perpetual unifoliate growth form resulting from an anisocotyledonous development (one-leaf plant) [4–6]. The herbaceous above-ground parts of mature plants primarily consist of a long cylindrical hypocotyl and a large glabrous macrocotyledon spanning areas of up to 0.5 m^2^ [7,8]. Under natural conditions, the excessive growth of the latter often causes hypocotyl overloading and collapse, finally resulting in the death of the plant [1,9]. On the other hand, *M. horsfieldii* shows striking morphological and structural alterations (i.e. distinct hypocotyl bending) in response to moderate water stress, which are fully recoverable after anew irrigation [7]. Generally, such phenomena are typical for desiccation-tolerant plants, which possess the ability to survive cellular water contents below 10% for several weeks or months until they rapidly regain normal function upon rehydration [10,11].

Poikilohydrous gesneriads of the European genus *Ramonda*, for example, display extensive leaf folding in order to limit light-induced cellular damage in the desiccated state [12,13]. Furthermore, studies on *Ramonda serbica* Panč. and *Ramonda nathaliae* Panč. et Petrov. also reported physiological adaptations to severe water stress [13–18]. Although they possess distinct growth forms and live in different climate zones, *Ramonda* species experience environmental conditions which are comparable with those of *M. horsfieldii* (i.e. shallow limestone soils, seasonal droughts in usually well-hydrated habitats and exposure to abiotic stresses) [12,13]. Moreover, several poikilohydrous gesneriads (i.e. *Boea hygrometrica*) also grow on rocky substrates and possess both physiological and structural adaptations to desiccation [19].

To date, detailed mechanical analyses of the main supporting structures of *M. horsfieldii*, the hypocotyls, are also lacking. In previous experiments, we qualitatively demonstrated that the posture and mechanical stability of *M. horsfieldii* are influenced by water status alterations (similar to poikilohydrous gesneriads), and also that they are unable to recover from relative water contents (RWCs) below 10% (unlike poikilohydrous gesneriads) [7]. However, its ability to withstand moderate water stress could possibly render *M. horsfieldii* an important model plant for studying the evolution of desiccation tolerance in Gesneriaceae. Therefore, we analysed the water-dependent changes in the anatomical, morphological and mechanical properties of greenhouse-cultivated *M. horsfieldii* plants by combining dehydration–rehydration experiments (DREs) with various imaging techniques and also with bending experiments and turgor pressure measurements, and compared the passive regeneration capacity of *M. horsfieldii* with that of its poikilohydrous relative *Ramonda myconi*.

## 2. Material and methods

### 2.1. Plant material

*Monophyllaea horsfieldii* plants were propagated by seeds and cultivated in a shaded tropical greenhouse chamber at an average temperature (*T*) of 25.7 ± 2.3°C and an average relative humidity (RH) of 61.9 ± 8.3% in the Botanic Garden Freiburg (for further details, see [7]). The initial stock of plants, from which the seeds were harvested, was donated by the Bonn Botanic Gardens (accession no.: 13885; IPEN: xx-0-BONN-13885; herbarium no.: 1727). Between experiments, the test plants were well watered every 2 days. Only mature plants, as indicated by flowering, with an average hypocotyl length and a macrocotyledon area of 10.5 cm and 214.2 cm^2^, respectively, were tested. *Ramonda myconi* (L.) Rchb. plants were purchased from Kaiserstühler Staudenhof Menton GdbR (Eichstetten, Germany) and kept in a temperate chamber at 11.0 ± 2.5°C and 64.4 ± 10.4% RH (average values) without shading. The verification of the correct plant species was made according to its flower morphology displaying mucronate stamen, which only occur in *R. myconi* (see electronic supplementary material, figure S3, and [20,21]). The diameters of their rosettes measured 12–15 cm and exhibited an average leaf length and width of 5.7 cm and 2.5 cm, respectively. The aforementioned values for *T* and RH represent the average cultivation conditions during the main test period (October/November 2015). As the cultivation conditions fluctuated within the main test period, it is important to mention that *M. horsfieldii* plants (as to their ecological requirements) have always experienced higher temperatures and lower light intensities than *R. myconi* plants.

### 2.2. Determination of relative water contents

In the course of anatomical tissue and pressure probe measurements, cylindrical hypocotyl sections (5 mm in length) were excised to determine RWCs, whereas circular macrocotyledon samples (8 mm in diameter) were punched out for RWC measurements accompanying the two-point bending experiments (see below). Detailed descriptions and the respective formula of the RWC determination are given in [7] and electronic supplementary material, appendix S1.

### 2.3. Dehydration–rehydration experiments

One day before each DRE, all plants were irrigated a last time to achieve identical starting conditions. Subsequently, they were passively dehydrated by slow soil drying due to evapotranspiration in consequence of the prevention of irrigation. The same procedure was applied in all DREs of this study, which only varied according to their dehydration durations between different experiments (for further details, see the descriptions below).

#### 2.3.1. Morphological changes in *Monophyllaea horsfieldii* hypocotyls

**Figure 1.**
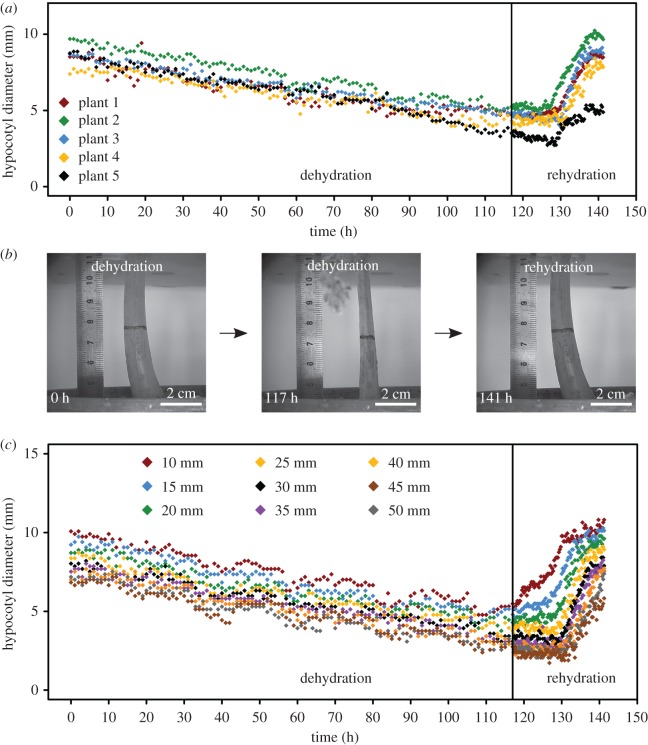
Changes of hypocotyl diameter in *M. horsfieldii* during a DRE. (*a*) The decrease and increase of the hypocotyl diameter at a marked position (30 mm above ground) of five different plants are shown in different colours. The majority of plants regain their original hypocotyl diameter after rehydration. (*b*) The three images exemplarily show the change of hypocotyl diameter in the aforementioned DRE. The hypocotyl shown belongs to plant two in (*a*). The time scale and scale bars are indicated. (*c*) The diameter changes along the hypocotyl of *M. horsfieldii* at 5 mm intervals are illustrated. Each colour represents one of nine different hypocotyl positions between 10 and 50 mm above ground.

Diameter changes of five intact hypocotyls were analysed in a 5-day dehydration and 1-day rehydration set-up (*T* = 24.7°C, RH = 65.3%, mean values of laboratory conditions). Single images were acquired from lateral perspectives throughout the whole DRE using USB microscope cameras (Conrad, Hirschau, Germany) with recording speeds of 1 frame h^−1^ and 12 frames h^−1^ for dehydration and rehydration phases, respectively. Afterwards, the diameters were measured framewise at a marked position mid-length of the hypocotyl (figure 1*b*) using ImageJ (v.1.50b, US National Institutes of Health, Bethesda, MD, USA) [22]. Additionally, the diameters of one plant were analysed at several hypocotyl heights (10–50 mm above ground at 5 mm intervals). Finally, dehydration and rehydration velocities were determined from diameter–time diagrams (slopes of the respective regression lines), whereas the initial, dehydrated and rehydrated hypocotyl diameters were used to calculate the percentage diameter reduction and recovery (for more details, see electronic supplementary material, appendix S1).

#### 2.3.2. Anatomical changes in *Monophyllaea horsfieldii* hypocotyls

Anatomical changes in various tissues were analysed in hypocotyl cross sections during a 9-day dehydration and 1-day rehydration experiment with a sampling rate of one plant per day (*T* = 17.3°C, RH = 77.5%, mean values of laboratory conditions). First, the RWC was measured (see §2.2) before the remaining hypocotyl was sectioned into slices, each of approximately 150 µm thickness, using a hand microtome (MT.5503, Euromex Microscopen, Arnhem, The Netherlands). Afterwards, five thin sections were placed on microscope slides and imaged using a stereo microscope (SXZ7 with DF PLAPO 1×^−2^ objective, Olympus, Tokyo, Japan) equipped with a digital camera (PL-D685C4, PixeLINK, Ottawa, Canada) and µScope software (v. 20.1, IMT i-Solution, Inc., Ottawa, Canada). At day 9, strongly dehydrated samples necessitated the production of slightly thicker sections (200–300 µm). Generally, the complete sampling and imaging procedure took 5–10 min. Finally, several cross-sectional parameters were measured with ImageJ along an initially determined measurement axis (see electronic supplementary material, figure S1).

#### 2.3.3. Two-point bending measurements

Two-point bending measurements were performed daily and in random order on 29 plants during a 5-day dehydration and 1-day rehydration experiment. First, RWC values were measured once per plant and day (see §2.2) prior to each bending test, which were performed using a 1 N force sensor (Burster 8510-5001, Burster Präzisionstechnik GmbH & Co. KG, Gernsbach, Germany), a linear motor (1624E012S, Faulhaber, Schönaich, Germany), a X–Y–Z-micromanipulator (MM33, Märzhäuser, Wetzlar, Germany) and a fixation system (see electronic supplementary material, figure S2). In particular, the force sensor and micromanipulator were controlled by a custom-made software (Pollination Simulation v. 1.3f, Technical Workshop, Institute of Biology II/III, University of Freiburg, Germany [23]) with a data acquisition rate of one value per 0.05 mm bending deflection. During all measurements, the plants were deflected to a maximum of 90 mm and loading forces were recorded simultaneously. However, when the piston connected to the sensor support noticeably slipped on or off the respective test plant, the recordings were stopped manually before reaching the maximum deflection. If plants were not mechanically testable due to severe water loss-induced slackening, their water status was re-established in the further course of the experiments. Between subsequent bending tests, all plants were stored at 22.9°C and 59.6% RH (mean values of laboratory conditions). The structural bending modulus for tapered beams was calculated as described in [24]; for more details, see also electronic supplementary material, appendix S1.

#### 2.3.4. Turgor pressure measurements

Tissue-specific turgor pressures of *M. horsfieldii* hypocotyls were quantified using the pressure probe method and the same equipment as described in [24]. Throughout a 10-day dehydration and 1-day rehydration set-up, turgor pressures and RWC values (see §2.2) were measured from one 5-cm-long hypocotyl sample, which was excised at mid-length and whose cutting surfaces were sealed with desiccator grease (Carl Roth GmbH & Co. KG, Karlsruhe, Germany). During each pressure probe measurement, turgor pressures were recorded multiple times over an insertion depth range of 25–2050 µm. Finally, tissue-specific correlations between turgor pressures and RWC values have been determined (here the assignment to specific hypocotyl tissues was made according to the insertion depths).

### 2.4. Quantification of the passive regeneration capability using a shrinking-and-swelling approach

*Monophyllaea horsfieldii* hypocotyls were cut into six 5-mm-long cylinders (one per treatment) to evaluate to what extent the rehydration process is governed by purely passive effects (passive water uptake and swelling of cells and tissues). Each sample was weighed with a high-precision balance (UMT2, high-precision balance, Mettler-Toledo, Greifensee, Switzerland) to determine its fresh weight, and subsequently dried at 80°C in a heating cabinet (TK/L4250, Ehret, Emmendingen, Germany): the first (second, third, fourth, fifth and sixth) sample of each hypocotyl was dried for 15 min (30 min, 1 h, 2 h, 4 h and 8 h, respectively). Subsequently, their weights were measured and the shrunken cylinders were incubated in distilled water for 24 h. After the careful removal of excess water with paper towels, the rehydrated weights were determined to quantify the regeneration capability (see electronic supplementary material, appendix S1). Identical measurements were performed on the leaf stalks of *R. myconi*, which represent different plant structures, but are also rod-shaped and functionally comparable to *M. horsfieldii* hypocotyls. The experiments have been replicated 10 times per species.

### 2.5. Statistics

Statistical analyses were performed with GNU R v.3.2.5, including the packages *car*, *psych* and *DescTool* [25–28]. Having checked the assumptions for normally distributed data (Shapiro–Wilk test) and homoscedasticity of the variances (Levene test) in advance, we analysed the non-parametric datasets using descriptive statistics (Med, median; IQR, interquartile range; Min, minimum; Max, maximum; *N*, sampling rate). Furthermore, Kruskal–Wallis tests (see two-point bending measurements, regeneration capability experiments and hypocotyl cell area analyses) were performed together with pairwise comparisons after Tukey and Kramer (Nemenyi *post hoc* testing) to test for significant differences. Correlations were calculated according to Pearson (*r*, parametric data) or Spearman (*ρ*, non-parametric data); for details, see [7]. Complete datasets are given in electronic supplementary material, appendix S2.

## 3. Results

### 3.1. Effect of water availability changes on morphological and anatomical properties of *Monophyllaea horsfieldii* hypocotyls

Our results show that the posture of *M. horsfieldii* is strongly affected by the plant's water status (figure 1). Dehydration experiments revealed water-dependent diameter reductions in the hypocotyl between 38% and 65% of the initial diameter, which were completely recoverable upon rehydration in four out of five plants (figure 1*a,b*). Moreover, detailed analyses along the hypocotyl of a single plant displayed a gradual diameter decrease with an average dehydration velocity of 0.03 mm h^−1^ independent from the location of measurement along the hypocotyl (figure 1*c* and table 1). On the other hand, rehydration velocities were approximately 10 times faster, revealing an acropetal diameter increase with basal hypocotyl regions rehydrating and swelling before the apical ones (figure 1 and table 1).

**Table 1.** Morphological changes in *M. horsfieldii* hypocotyls.

hypocotyl diameter change	median	IQR	minimum	maximum	*N*
initial hypocotyl diameter (mm)	8.69	0.23	7.39	9.69	5
diameter of the dehydrated hypocotyl (mm)	4.71	0.54	3.52	5.26	5
diameter of the rehydrated hypocotyl (mm)	8.49	0.91	4.89	9.66	5
dehydration velocity (mm h^−1^)	−0.03	0.01	−0.04	−0.03	5
rehydration velocity (mm h^−1^)	0.28	0.09	0.14	0.34	5
diameter reduction (%)	55.3	1.67	40.3	58.5	5
diameter recovery (%)	99.7	1.00	55.9	106.2	5

**Figure 2.**
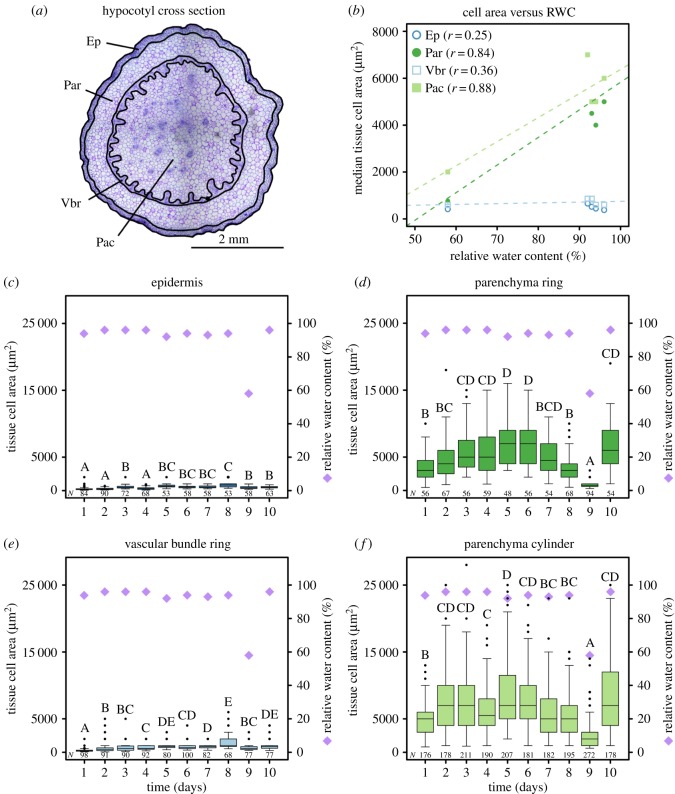
Anatomical changes in the tissues of *M. horsfieldii* hypocotyls during a DRE. (*a*) From outside to inside, the hypocotyl consists of four tissue regions: epidermis (Ep), peripheral parenchyma ring (Par), vascular bundle ring (Vbr) and central parenchyma cylinder (Pac). (*b*) In contrast to the cells of the epidermis and the vascular bundle ring, those of the parenchymatous tissues show a strong correlation between RWC and median cell area (Ep, blue open circles; Par, green circles; Vbr, light-blue open squares; Pac, light-green squares). Pearson's correlation coefficients (*r*) are given in parentheses. (*c–f*) The parenchymatous tissues exhibit the highest cell area changes during dehydration (days 1–9) and rehydration (days 9–10) of the hypocotyl (green and light-green boxplots), whereas those of the epidermis and vascular bundle ring hardly change at all (blue and light-blue boxplots). The RWC alteration is indicated by purple diamonds. Significant changes are marked by capital letters above individual boxplots. The corresponding sample sizes (*N*) are indicated below each boxplot.

Anatomically, *M. horsfieldii* hypocotyls comprise epidermal (Ep: epidermis, typically six cell layers thick, median value), parenchymatous (Par: peripheral parenchyma ring, typically six cell layers thick; Pac: central parenchyma cylinder, typically 54 cell layers thick; median values) and vascular (Vbr: vascular bundle ring, typically seven cell layers thick, median value) tissue layers, which are arranged in four distinct areas (figure 2*a* and table 2 for precise radial thicknesses). These anatomical properties of tissues comprising the *M. horsfieldii* hypocotyls again exhibited considerable alterations in response to changing water availabilities. More precisely, we found that the median cell areas of the majority of tissues are positively correlated with the RWC of the hypocotyl (Pearson's product–moment correlation, Ep: *r* = 0.25; Par: *r* = 0.84; Vbr: *r* = 0.36; Pac: *r* = 0.88; figure 2*b*). Here, the parenchymatous tissues (Par and Pac) exhibited the highest correlations between the median cell areas and their respective RWC values (figure 2*b*). Consistently, the cell areas of epidermal and vascular bundle cells changed — both absolutely and relatively — less than those of parenchymatous cells (figure 2*c–f* and table 3). Among all hypocotyl tissue regions, the cells of the peripheral parenchyma ring displayed the highest cell area alterations with varying hydration status (figure 2*d* and table 3).

**Table 2.** Average radial thicknesses of tissues in *M. horsfieldii* hypocotyl cross sections.

average tissue radial thickness (mm)	median	IQR	minimum	maximum	*N*
epidermis, Ep	0.12	0.03	0.08	0.24	50
parenchyma ring, Par	0.24	0.04	0.16	0.46	50
vascular bundle ring, Vbr	0.14	0.04	0.10	0.29	50
parenchyma cylinder, Pac (radius)	1.08	0.16	0.82	1.96	50

**Table 3.** Tissue-specific cell area changes in *M. horsfieldii* hypocotyls in response to RWC alterations. The absolute change is the difference between the average median cell area (MCA) at RWCs >90% and the MCA at lowest RWC, whereas the relative change is the ratio of the absolute change and the average MCA at RWCs >90%.

tissue	average MCA at RWCs >90% (µm^2^)	MCA at lowest RWC (µm^2^)	absolute change (µm^2^)	relative change (%)
Ep	511	399	112	21.9
Par	5250	775	4475	85.2
Vbr	739	590	149	20.2
Pac	5750	2000	3750	65.2

It is important to mention that the nature of RWC fluctuations during the anatomical measurements (as shown in figure 2*c–f*) likely results from both the irrigation schedule and sampling intervals. In particular, passive soil drying caused the dehydration of the moist soil surrounding the freshly irrigated test plants (see §2.3), leading to small RWC fluctuations within the first 8 days (figure 2*c–f*). Once the soil was dry, a distinct RWC drop became visible at day 9 (figure 2*c–f*) whose intensity probably arose from the small sampling rate of one plant per day in these combined experiments.

### 3.2. Effect of changes in water status on the biomechanical properties of *Monophyllaea horsfieldii* hypocotyls

**Figure 3.**
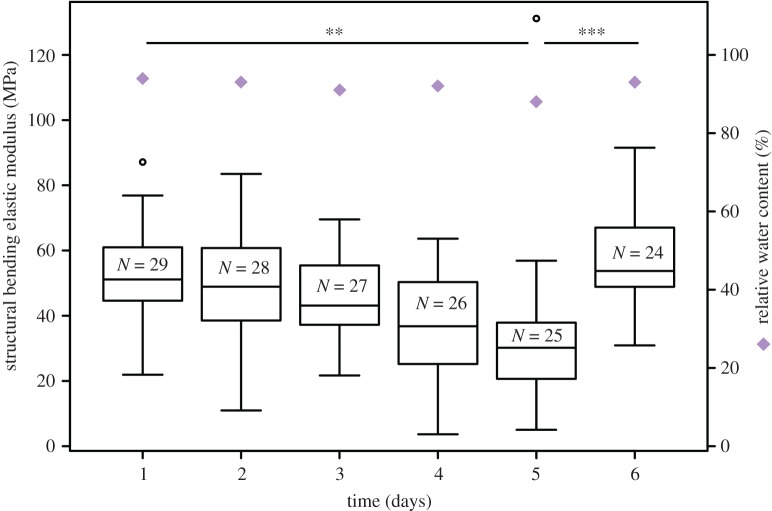
Change in the structural bending modulus of *M. horsfieldii* hypocotyls during a DRE. Illustration of the changes in structural bending modulus during DRE according to the alterations of the RWC (black circles). The significant reduction of the structural bending modulus during dehydration (days 1–5) and its recovery after rehydration (days 5–6) are highlighted. The RWC alteration is indicated by purple diamonds. *N*, sample size; **p < 0.01; ***p < 0.001.

Two-point bending tests performed in combination with DREs demonstrated that already a small RWC drop from 93% to 82% significantly reduced the structural bending modulus (Kruskal–Wallis test, $\chi_{\left(5\right)}^{2}=33.72,$ *p* < 0.001, pairwise Wilcoxon rank sum *post hoc* testing; figure 3; electronic supplementary material, appendix S3 and S4). In particular, the measured decrease of approximately 20 MPa corresponds to a reduction in the structural bending modulus by nearly 40%. Only 24 h upon rehydration, however, *M. horsfieldii* hypocotyls had fully recovered their postures and biomechanical properties after the applied moderate water stress (figure 3; electronic supplementary material, appendix S3).

**Figure 4.**
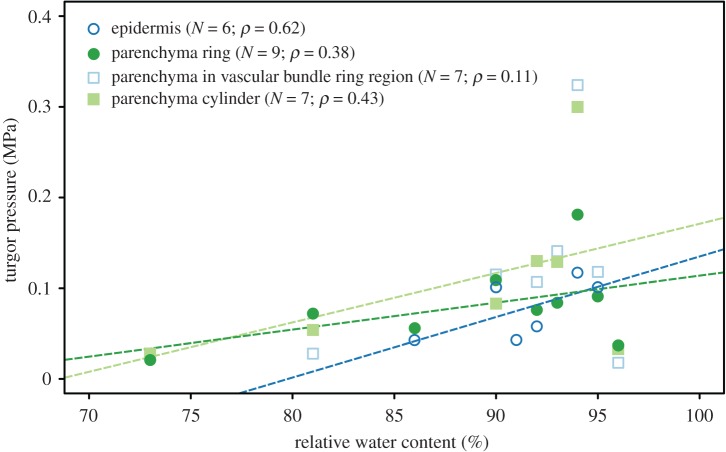
Relationship between turgor pressures and RWCs in different tissues of *M. horsfieldii* hypocotyls. In the cells of the epidermis (blue open circles), the parenchyma ring (green circles) and the parenchyma cylinder (light-green squares), the turgor pressure correlates positively with the RWC (Spearman's correlation coefficients are given). Only the parenchyma cells in the vascular bundle ring region (light-blue open squares) show no correlation between the RWC and the turgor pressure. The *ρ* values are given to show a general trend. However, it has to be kept in mind that for a reliable statistical analysis, much larger sample sizes would be necessary. *N*, sample size; *ρ*, correlation coefficient.

Additional turgor pressure measurements revealed that the internal cell pressures of six epidermal cells (range 0.04–0.12 MPa), nine parenchyma ring cells (range 0.02–0.18 MPa), seven cells from parenchyma in the vascular bundle ring region (range 0.02–0.32 MPa) and seven cells from the central parenchyma cylinder (range 0.03–0.30 MPa) are positively correlated with corresponding RWCs in three of the four tissue regions (Spearman's rank-order correlation, Ep: *ρ* = 0.62; Par: *ρ* = 0.38; Pac: *ρ* = 0.43; figure 4). Contrastingly, a correlation between parenchymatous cells in the region of the vascular bundle ring and corresponding RWC values was not found (Spearman's rank-order correlation, Vbr: *ρ* = 0.11; figure 4).

### 3.3. Passive regeneration capability of homoiohydrous and poikilohydrous gesneriads using a shrinking-and-swelling approach

**Figure 5.**
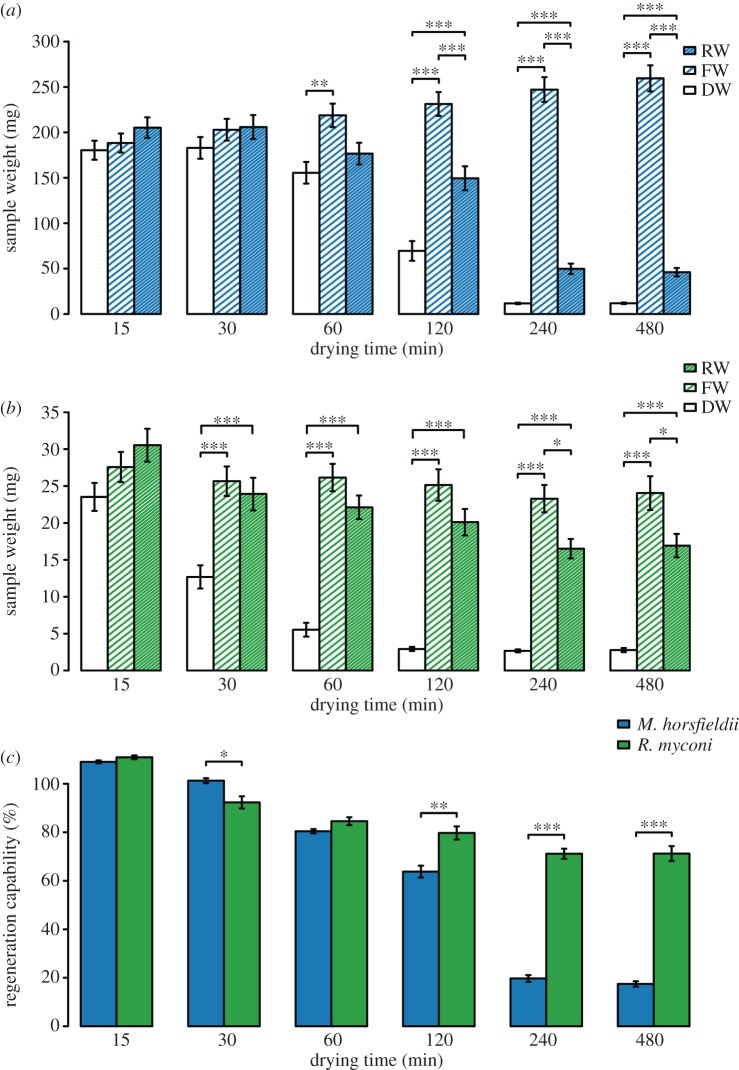
Comparison of the regeneration capacities of *M. horsfieldii* hypocotyl and *R. myconi* leaf stalk segments after different drying durations. (*a*,*b*) The regeneration capability of *M. horsfieldii* hypocotyl segments is considerably less than that of *R. myconi* leaf stalk segments after different drying durations. (*c*) Comparisons of *M. horsfieldii* and *R. myconi* regeneration capacities after different drying durations indicate different amounts of passively functioning adaptations. Values are given as means ± standard errors; *N* = 10; *p < 0.05; **p < 0.01; ***p < 0.001; DW, dry weight; FW, fresh weight; RW, rehydrated weight.

Finally, we compared the passive regeneration capacities of *M. horsfieldii* hypocotyl sections and *R. myconi* leaf stalk sections in shrinking-and-swelling experiments. Short drying durations of up to 30 min did not affect the sample weights of *M. horsfieldii* hypocotyl sections, whereas increased drying durations of 1 h led to significant weight differences between fresh and dry weight and reduced the regeneration capability by 20–30% (Kruskal–Wallis test, 1 h: $\chi_{\left(2\right)}^{2}=10.20,$ *p* < 0.01, Nemenyi *post hoc* testing; figure 5*a*,*c*). Longer drying periods resulted in significant differences between the weights of freshly cut and rehydrated samples, leading to a minimum regeneration capability of *M. horsfieldii* hypocotyls of 17.05% after a drying period of 8 h (Kruskal–Wallis test, 2 h: $\chi_{\left(2\right)}^{2}=22.58,$ *p* < 0.001; 4 h: $\chi_{\left(2\right)}^{2}=25.81,$ *p* < 0.001; 8 h: $\chi_{\left(2\right)}^{2}=25.81,$ *p* < 0.001; Nemenyi *post hoc* testing; figure 5*a*,*c*).

In contrast to *M. horsfieldii*, the leaf stalk sections of *R. myconi* showed first significant differences between fresh and dry sample weights already after 30 min of drying (Kruskal–Wallis test, 30 min: $\chi_{\left(2\right)}^{2}=15.30,$ *p* < 0.001; Nemenyi *post hoc* testing; figure 5*b*). As long as the drying duration was equal to or less than 2 h, fresh and rehydrated sample weights did not differ significantly and the respective regeneration capability values remained comparatively high, i.e. between 83% and 89% (Kruskal–Wallis test, 15 min: $\chi_{\left(2\right)}^{2}=5.30,$ *p* > 0.05; 30 min: $\chi_{\left(2\right)}^{2}=15.30,$ *p* < 0.001; 1 h: $\chi_{\left(2\right)}^{2}=20.05,$ *p* < 0.001; 2 h: $\chi_{\left(2\right)}^{2}=20.19,$ *p* < 0.001; Nemenyi *post hoc* testing; figure 5*b*,*c*). Only extreme drying durations (4 and 8 h) led to an incomplete recovery of sample weights after rehydration (Kruskal–Wallis test, 4 h: $\chi_{\left(2\right)}^{2}=22.00,$ *p* < 0.001; 8 h: $\chi_{\left(2\right)}^{2}=21.34,$ *p* < 0.001; Nemenyi *post hoc* testing; figure 5*b*). Nevertheless, we observed regeneration capacities of *R. myconi* leaf stalk segments after 8 h of drying, which were 4.2 times higher than those of *M. horsfieldii* hypocotyl sections (figure 5*c*).

## 4. Discussion

### 4.1. Effect of water availability changes on morphological and anatomical properties of *Monophyllaea horsfieldii* hypocotyls

Anhydrobiosis is a strategy found in all organismal kingdoms allowing the survival of prolonged periods of severe droughts by drying to RWCs below 10% [29]. Among higher plants, anhydrobiotes are termed ‘resurrection plants’ and have evolved independently in multiple lineages [30]. A high occurrence of desiccation-tolerant plants is present in the Gesneriaceae, which may be linked to their presence on limestone-based soils that are rich in calcium [19]. Calcium is an essential plant macronutrient with key structural and signalling roles, which interacts together with abscisic acid in the regulation of dehydration-induced gene expression in various resurrection plants such as *B. hygrometrica* or *Craterostigma plantagineum* [31–35]. Often, poikilohydrous gesneriads possess both structural and physiological adaptations to desiccation. Species of the genera *Ramonda* and *Haberlea*, for example, increase their sucrose and raffinose levels during dehydration to protect their plasma membranes from critical injuries [17]. Moreover, *R. serbica* accumulates ascorbate and glutathione to inhibit oxidative effects during desiccation [18]. Further information on the roles of sugars, antioxidants, reactive oxygen species scavenging enzymes and additional protective proteins are reviewed in [19].

In contrast to these physiological adaptations, the knowledge on structural adaptations of poikilohydrous gesneriads is scarce. Adaptive postural changes in response to water deficits have been reported for the leaf architectures of *B. hygrometrica*, *R. serbica* and *R. nathaliae* [13,19]. Neither a distinct shrinkage nor a curling towards the adaxial surface of the leaf was visible in our DREs on *M. horsfieldii*. A protection against photoinhibition and reactive oxygen species production by morphological means is therefore highly unlikely (cf. [19]). Furthermore, previous qualitative dehydration–rehydration analyses on *M. horsfieldii* revealed that severe water loss leads to irreparable damages (probably due to rupturing and plasmolytic disintegration of the plasma membrane from the cell wall) and causes a collapse of the above-ground plant parts, entailing the death of the plant [1,7,10,11,36,37]. However, the observed reversible reductions of up to 65% of the initial hypocotyl diameters in *M. horsfieldii* during moderate water stress clearly suggest a connection between the posture and the actual water balance of the plant. We therefore assume that specific hypocotyl tissues are structurally very resilient and are capable of repeatedly losing and regaining considerable amounts of water (see §3.1 and the discussion of the anatomical analyses below). Further differences between the directionalities (uniform shrinkage versus acropetal swelling) and the velocities (time scales) of dehydration and rehydration processes probably trace back to different physical principles (i.e. evapotranspiration by slow diffusive processes during dehydration versus root water uptake and vascular water transport by fast volume transport processes during rehydration) [38,39].

Apart from the aforementioned postural changes, structural adaptations to severe water stress have also been reported on cell and tissue levels. More precisely, the desiccation-induced damage on the plasma membranes in some poikilohydrous plant species is limited by the preservation of a high cell wall flexibility during dehydration, which enables an extensive wall folding to avoid destructive load peaks [40–42]. Our analyses on *M. horsfieldii* revealed neither tissue- nor cell-specific protection mechanisms, such as cell wall folding.

### 4.2. The impact of water status on the bending resistance of *Monophyllaea horsfieldii* hypocotyls

However, the tissue arrangement found in the hypocotyl is ‘meaningful’ for a structure in which the predominant natural loading type is bending. Here, the epidermis functions not only as a protective and mechanically stabilizing boundary tissue but also as an antagonist (in tension) to the pressures occurring from the inner (parenchymatous) tissues finally forming an outer ‘tension bracing’ [43,44]. Analogously, the vascular bundle ring acts as a second internal reinforcement contributing to a higher bending stiffness of the hypocotyl by possessing water-conducting cells with lignified walls [7,45,46]. On the one hand, this helps to maintain or to rapidly regain turgor pressure in the adjacent parenchyma cells. On the other hand, the vascular bundle ring not only represents a stiffening tissue but may also act as an internal ‘tension bracing’ interacting with the turgor pressures of the surrounding parenchyma tissues. Moreover, cell wall thickness and degree of lignification strongly affect stiffness and deformability [47]. Therefore, it is not surprising that—in our experiments—thick-walled and, hence, stiffer epidermal cells and lignified vascular bundle cells hardly react to water changes, whereas compliant thin-walled parenchyma cells deformed to a larger extent [47–49].

Furthermore, the positive correlations between the median cell areas of parenchyma tissues and their respective RWC values clearly suggest that the mechanical stability of *M. horsfieldii* is mainly turgor-driven. Thus, we hypothesize that turgor alterations at cellular level determine the macroscopic hypocotyl shape and stability at tissue level. At high water availability, the highly pressurized central parenchyma cylinder expands and presses against the mechanically stable vascular bundle ring. Additionally, the peripheral parenchyma ring inflates with increasing turgor and can be considered as a kind of ‘pressurized lining’ between the vascular bundle ring and the epidermis [50–52]. Besides that, epidermal and vascular tissues are known to provide substantial longitudinal bending stiffness in non-lignified plant axes, while turgor pressures simultaneously apply considerable tensile stresses onto the cell walls within parenchymatous and epidermal tissues, thereby hindering stem buckling under axial and lateral loading conditions [43,44,50–53]. Therefore, increasing turgor pressures (and RWCs) lead to an overall improvement of the mechanical stability in *M. horsfieldii* hypocotyls [43,44]. At low water availability, parenchymatous tissues shrink dramatically as RWCs decrease, and lose their function as pressurized lining providing progressively lesser protection against bending and global buckling. Owing to the tissue arrangement in hypocotyls, lower water contents allow for higher flexibility and larger deflections of the hypocotyls [43,44,52,54] (see also electronic supplementary material, appendix S2).

In addition to that, we demonstrate that already small water content reductions of approximately 10% led to a 50% decrease in the structural bending moduli, and that the RWCs of cells from non-lignified hypocotyl tissues (i.e. epidermal and parenchymatous tissues) are positively correlated to their respective turgor pressures. Coherently, wilting experiments on petioles of *Caladium bicolor* revealed a significant decrease in flexural rigidity caused by a water loss-induced reduction in the mechanical efficiency of collenchyma fibres and by turgor loss of parenchyma cells [24]. Further studies on potato tuber parenchyma revealed a linear dependence of the Young's modulus on their internal cell pressures [55,56].

### 4.3. Quantification of the passive regeneration capability in *Monophyllaea horsfieldii* using a shrinking-and-swelling approach

Often, poikilohydrous gesneriads, such as *B. hygrometrica*, *R. serbica* and *R. nathaliae*, exhibit pronounced shrinkage and leaf curling movements towards their adaxial lamina surface during dehydration and thus can limit light-induced damage of the photosynthetic apparatus [13,19]. Some of these protective plant movements additionally function in detached leaves hinting at purely structure-based function principles [19]. *Monophyllaea horsfieldii* also shows reversible postural changes upon dehydration in terms of moderate water stress conditions [7]. However, comparative analyses on the passive regeneration capacities of *M. horsfieldii* and *R. myconi* revealed that only 17% of the regeneration capability in *M. horsfieldii* hypocotyls can be linked to adaptations functioning passively, while leaf stalk sections of *R. myconi* passively regenerated 72% of their initial weights after prolonged drying. Thus, we assume that *R. myconi*—probably similar to its Balkan relatives—possesses both passively functioning (i.e. passive shrinking-and-swelling processes) and physiologically driven structural adaptations (i.e. cell wall folding) to avoid fatal cell damage during (severe) dehydration and rehydration [13,57–60].

## 5. Conclusion

Our experiments support the assumption that the overall mechanical stability of *M. horsfieldii* is strongly affected by cell and tissue pressures which themselves are markedly influenced by the actual water status of the plant. Structural adaptations, i.e. in the cell walls, causing specific swelling and shrinking effects are thought to play a minor role in this species. However, further comparative studies focusing on the quantitative comparison of *M. horsfieldii* (or a similar species) and a second, desiccation-tolerant gesneriad (e.g. *Ramonda*, *Haberlea* and *Boea*) may reveal novel structural adaptations to poikilohydry.

## Supplementary Material

Supplementary Methods & Figures

## Supplementary Material

Datasets

## Data accessibility

The datasets supporting this article have been uploaded as part of the electronic supplementary material.
