# Supplementary material for: How water availability influences morphological and biomechanical properties in the one-leaf plant Monophyllaea horsfieldii
Source: R Soc Open Sci. 2018 Jan 3;5(1):171076. doi: 10.1098/rsos.171076 (PMC5792897; doi:10.1098/rsos.171076)
Supplement: Supplementary Methods & Figures [file rsos171076supp1.doc]

**Kampowski *et al*. 2017 – How water availability influences morphological and biomechanical properties in the one-leaf plant *Monophyllaea horsfieldii***

**Supplementary Methods**

**Determination of relative water contents (RWCs):**

Fresh weights (FWs), turgescent weights (TWs) and dry weights (DWs) of all samples have been determined as described in detail in Ref [1]. Here, FW relates to the weight which is measured in the initial step of each RWC measurement during the dehydration-rehydration experiment (DRE) independent from the time of sampling. Therefore, FW does not necessarily imply a well-hydrated state of the test plant. RWCs were calculated using the following equation:


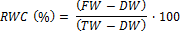


**Determination of hypocotyl diameter reduction and hypocotyl diameter recovery:**

First, the initial (IHD), dehydrated (DHD) and rehydrated (RHD) hypocotyl diameters have been determined from single frames acquired during a DRE using ImageJ (for version see section 2.3.1 in the main document). The percentage reduction and recovery of the hypocotyl were calculated using the following equations:

| 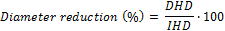 |  |
| --- | --- |
| 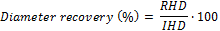 |  |

**Determination of tissue cell areas and average radial tissue thicknesses during a DRE:**

In each cross-section, all cells intersected by the measurement axis were analysed as to their cell areas (see Fig S1). Moreover, the average radial tissue thicknesses were determined along the measurement axis which add up to the radius of a virtual circle fitting the cross-section under inspection (see Fig S1B).

## **Two-point bending measurements at varying water contents:**

The structural bending elastic modulus for tapered beams were calculated using the following equation:

| 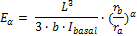 |  |
| --- | --- |

with *b* being the slope of the displacement-force diagram, *I_basal_* being the basal axial second moment of area for samples with a constant cross-section and *L* being the distance between the points of fixation and force application. The second term is an expansion of the standard bending modulus equation accounting for the hypocotyl tapering with *α* being the tapering mode and *r_a_* and *r_b_* being the apical and basal hypocotyl equivalent radii, respectively (see Appendix S1, Eq.1 and Eq.2 in [2]). The use of the above mentioned equation is only valid for straight beams. Therefore, *M. horsfieldii* test plants were pre-selected for R/D >> 8 (ratio of curvature radius and maximum hypocotyl diameter) allowing for the retention of the straight beam equation [3]. Additionally, plants were selected for their L/D-ratios (ratio of length and maximum diameter of the hypocotyl), since the influence of shear forces decreases with increasing L/D-ratios [4]. In summary, R/D- and L/D-ratios were calculated in advance from the radii of curvature, the hypocotyl lengths and the maximal hypocotyl diameters which were either measured from lateral view digital images using ImageJ (for version see section 2.3.1 in the main document) or directly at the plant using a calliper.

## **Quantification of the regeneration capability using a shrinking-and-swelling approach:**

The regeneration capability of *Monophyllaea horsfieldii* and *Ramonda myconi* have been calculated using the following equation:

| 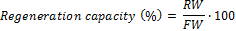  with *FW* and *RW* being the fresh and rehydrated sample weights, respectively. |  |
| --- | --- |

**Supplementary Figures**

**
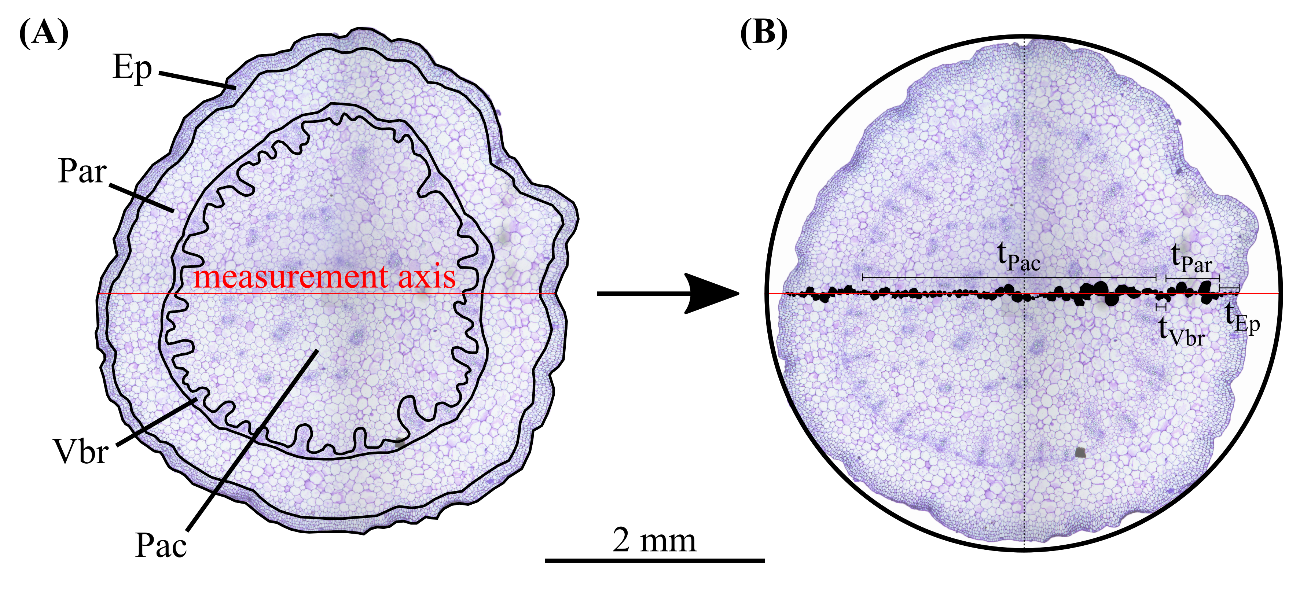
**

**Fig S1. Determination of tissue cell areas and average radial tissue thicknesses.** (A) LM micrograph of a *M. horsfieldii* hypocotyl section with highlighted tissue borders for better visibility. Areas were measured for all cells that are intersected by the measurement axis. (B) Example of tissue cell area and tissue thickness measurements along the previously determined measurement axis. Ep, epidermis; Pac, central parenchyma cylinder; Par, peripheral parenchyma ring; t_Ep_, thickness of epidermis; t_Pac_, thickness of central parenchyma cylinder; t_Par_, thickness of peripheral parenchyma ring; t_Vbr_, thickness of vascular bundle ring; Vbr, vascular bundle ring.


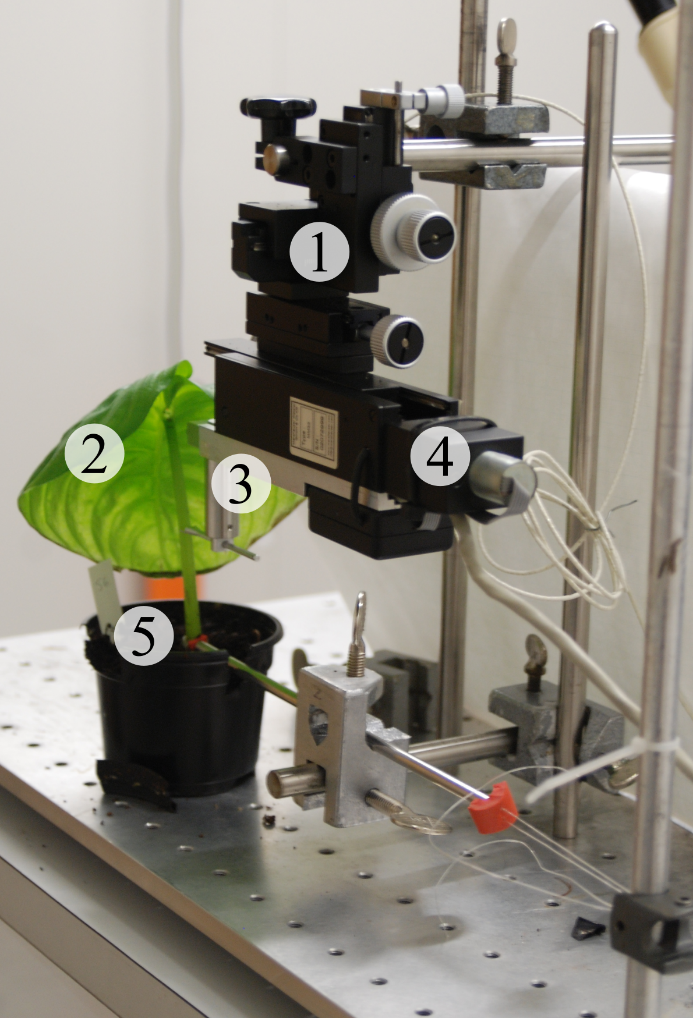


**Fig S2. Two-point-bending measurement setup.** The fixed *M. horsfieldii* hypocotyl (2, 5) can be displaced by an apparatus containing a 1-N force transducer (3), a linear motor (4) and a XYZ-micro-manipulator (1).


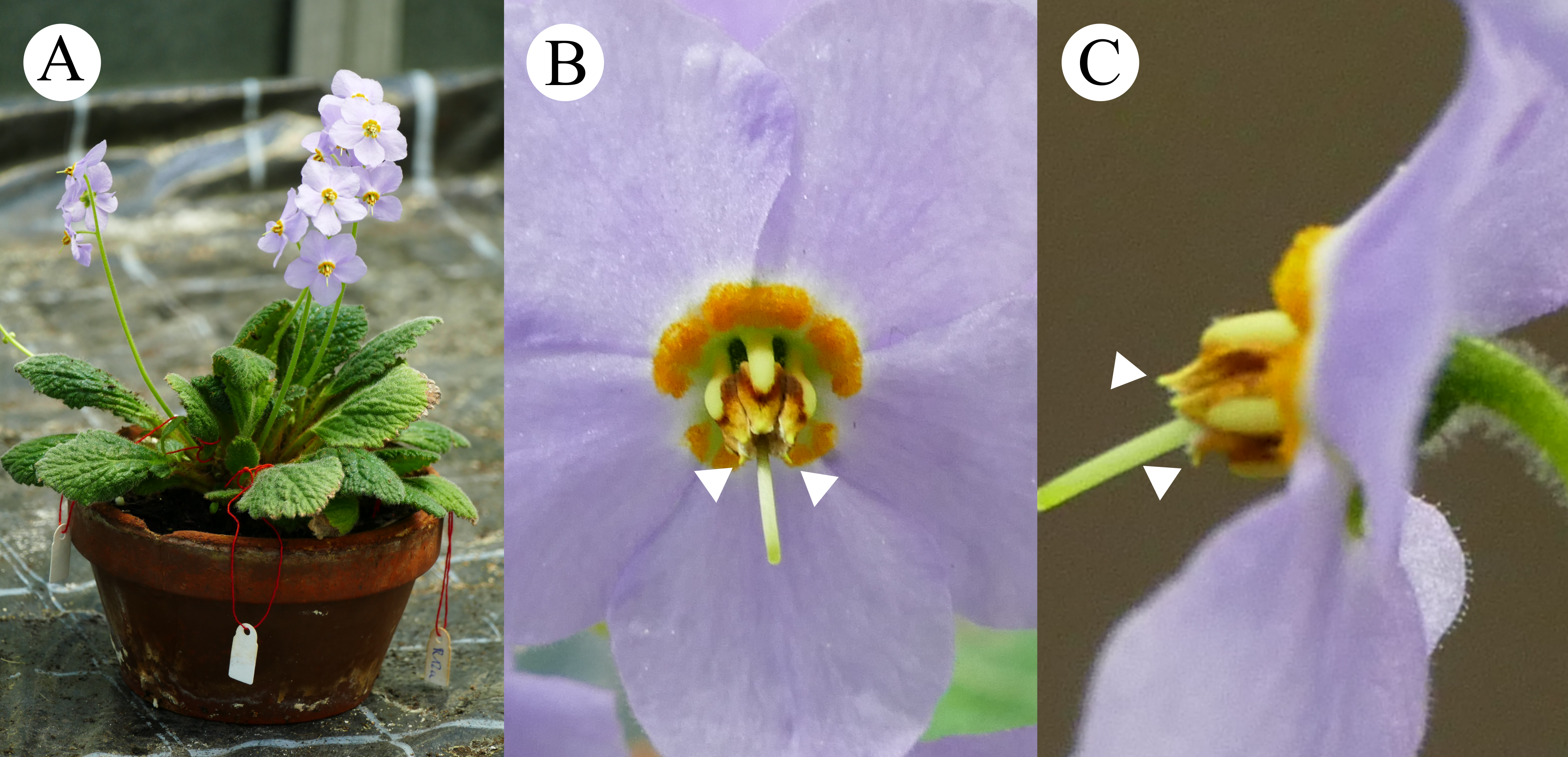


**Fig S3. Plant species identification according to the flower morphology of *Ramonda* test plants.** (A) Overview of potted *Ramonda* test plant. (B, C) Close-ups of the flower of *R. myconi* plant from frontal and lateral views, respectively, showing mucronate stamen (arrow), which neither occur in other *Ramonda* species nor hybrids [5, 6].

**References**

1. Kampowski T, Mylo MD, Speck T, Poppinga S. 2017 On the morphometry, anatomy and water stress behaviour of the anisocotyledonous *Monophyllaea horsfieldii* (Gesneriaceae) and their eco-evolutionary significance. *Bot. J. Linn. Soc.* , (accepted).

2. Caliaro M, Schmich F, Speck T, Speck O. 2013 Effect of drought stress on bending stiffness in petioles of *Caladium bicolor* (Araceae). *Am. J. Bot.* **100**, 2141–2148. (doi:10.3732/ajb.1300158)

3. Young WC, Budynas RG. 2002 *Roark’s Formulas for stress and strain*. Seventh edition. New York: McGraw-Hill Education Ltd.

4. Vincent J. 1992 Plants. In *Biomechanics - Materials: a practical approach* (ed J Vincent), pp. 165–191. IRL Press at Oxford University Press.

5. Tutin TG, Heywood VH 1972 *Flora Europaea Vol. 3*. 1^st^ Edition, p.285. Cambridge: Cambridge University

Press.

6. Jäger EJ, Ebel F, Hanelt P, Müller G 2016 *Rothmaler - Exkursionsflora von Deutschland: Krautige Zier-*

*und Nutzpflanzen*. 5th Edition, p.481. Berlin-Heidelberg: Springer.
